# Sea star *Henricia spiculifera* (Clark, 1901) in the northwestern Pacific: one species or three?

**DOI:** 10.7717/peerj.3489

**Published:** 2017-06-22

**Authors:** Anton Chichvarkhin

**Affiliations:** National Scientific Center of Marine Biology, Far Eastern Branch of Russian Academy of Sciences, Vladivostok, Russia; Far Eastern Federal University, Vladivostok, Russia

**Keywords:** Asteroidea, Echinodermata, Echinasteridae, New species, Sea of Japan, Kamchatka, Kommanders Islands

## Abstract

Three species of the sea stars are reported from the waters of the northwestern Pacific. These species were referred by earlier authors as *Henricia spiculifera* or* H. leviuscula spiculifera*. Two of them, *H. lineata* and *H. uluudax*, were recently described from the Aleutian Islands. These species are reported for the first time from the western Pacific (southeastern Kamchatka shore, Commander Islands, and the northern Kurile Islands). The third species, *H. olga* sp. n. is herein described from the northern Sea of Japan. It is very likely that similar sea stars recorded in Yellow Sea and the southern Kurile Islands belong to *H. olga* sp. n. These three species are a part of a phylogenetic clade within the subgenus *Setihenricia*, which also includes *H. sanguinolenta, H. multispina,* and several undescribed species occurring in the northeastern Pacific.

## Introduction

The sea stars studied here belong to the genus *Henricia* Gray, 1840 of the family Echinasteridae (Asteroidea, Spinulosida). The systematics of this taxon is poorly developed despite their abundance and wide distribution, in particular, in the northern Pacific (Verrill, 1909; Verrill, 1914; Fisher, 1911; Fisher, 1928; Fisher, 1930; Hayashi, 1940; Shin & Rho, 1996; Djakonov, 1961; Lambert, 2000; Clark & Jewett, 2010; Jewett et al., 2015; Chichvarkhin, 2017; Chichvarkhin & Chichvarkhina, 2017a; Chichvarkhin & Chichvarkhina, 2017b; Chichvarkhin, Chichvarkhina & Wakita, in press). *Henricia spiculifera* (Clark, 1901) was rather frequently reported from the northern Pacific by many authors as a distinct species or as an infraspecific form of *H. leviuscula* (Stimpson, 1857). Its distribution ranges from the Salish Sea, Puget Sound to the Yellow Sea (Clark, 1901; Verrill, 1914; Fisher, 1930; Hayashi, 1940; Djakonov, 1961; Shin & Rho, 1996; Lambert, 2000; Xiao, Liao & Liu, 2011). Many individuals of *Henricia* were found sitting on the sponges presumably filtering phytoplankton using water currents generated by the sponge colony (Rasmunsen, 1965). This species was easily distinguished from the other *Henricia* species with its fine slender spines possessing three or four long apical thorns (Djakonov, 1961; Xiao, Liao & Liu, 2011). Few other *Henricia* species possess similar spines (e.g., *H. densispina* (Sladen, 1878) and *H. sanguinolenta* (Müller, 1776)), but their spines are more thick and robust, and possess 4–9 thorns (Madsen, 1987; Xiao, Liao & Liu, 2011; Chichvarkhin & Chichvarkhina, 2017b). Recently, Clark & Jewett (2010) suggested that the combinations *H. spiculifera* and *H. levuiscula spiculifera* were used in the literature for several distinct species including *H. multispina* Fisher, 1910 (which they resurrected) and several undescribed entities; *H. lineata* Clark & Jewett, 2010 and *H. uluudax* Clark & Jewett, 2010 belonging to this group were described, while the ‘parent’ *H. spiculifera* was considered a *nomen inquirendum* because its type specimen was presumably lost, and more than one undescribed species belonging to the fine-spined group are known from its type locality according to DNA sequence data (Clark & Jewett, 2010).

Here, I present three distinct species from Russian waters of the Pacific, that were formerly identified and referred to as *H. spiculifera.* Since *H. spiculifera* is recognized as an invalid name (Clark & Jewett, 2010), all reported species are new records for the waters of the northwestern Pacific.

## Material and Methods

**Figure 1:**
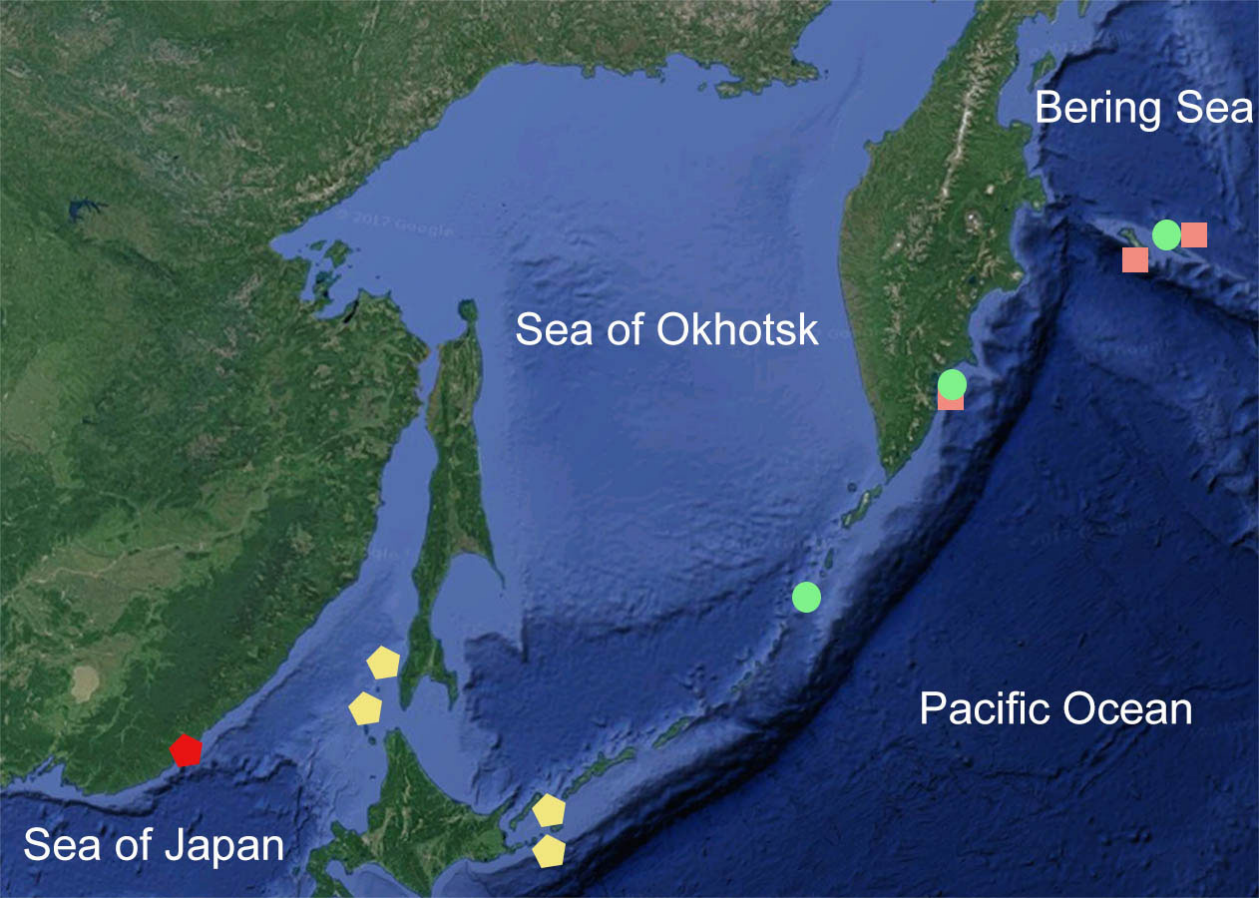
Surveyed area map. Pentagons, *Henricia olga* sp. n. (red, type locality; yellow, hypothesized *H. olga*); squares, *H. lineata*; circles, *H. uluudax*. Map data: ©2017 Google.

Observations and sample collections were taken by SCUBA-diving in 2014 through 2017 in Rudnaya Bay of the Sea of Japan and in the Gulf of Avacha (Avacha Bay and Starichkov Is.), Kamchatka (Fig. 1). Underwater images were taken with a Nikon D810 camera equipped with Nikkor 105/2.8G lens and appropriate Sea & Sea underwater housing. The other images were taken with a Nikon D810 or D7000 cameras and a Nikkor 60/2.8 lens. The specimens are in 96% ethanol and deposited in the Museum of National Scientific Center of Marine Biology, Russian Academy of Sciences, Vladivostok. Skeletal plates and spines were denuded using 5–15% sodium hypochlorite solution. Scanning electron images of the spines were obtained using Zeiss Sigma and Zeiss Evo electron microscopes after carbon coating. Other studied specimens are preserved in the collections of Zoological Institute of Russian Academy of Sciences, St. Petersburg, Russia (ZIN), and National Science Center of Marine Biology, Russian Academy of Sciences (MIMB), and Hokkaido University, Sapporo, Japan.

DNA was extracted using the Diatom™ DNA Prep 100 kit (Isogene Lab, Moscow, Russia) according to manufacturer’s protocol. Partial sequence of mitochondrial 16S rRNA gene (16S) was used in this study. The author successfully used this marker earlier for delimitation of *Henricia* species (Chichvarkhin, Chichvarkhina & Wakita, in press). The primers used to amplify that fragment (Palumbi, 1996) were used here to amplify the region of interest. The master mix for each sample was prepared using 34.75 µL H_2_O, 5.00 µL PCR Buffer (Evrogen, Moscow), 5.00 µL 25 mM MgCl_2_, 1.00 µL 40 mM dNTPs, 1.00 µL 10 mM primer 1, 1.00 µL primer 2, 0.25 µL 5 mg/mL Taq, and 1.00 µL extracted DNA. Reaction conditions were an initial denaturation for 3 min at 95 °C, 39 cycles of (1) denaturation for 45 s at 94 °C, (2) annealing for 45 s at 50 °C, and (3) elongation for 2 min at 72 °C, and a final elongation for 10 min at 72 °C. PCR products yielding bands of ca. 600 b.p. were purified using ethanol precipitation. Sequencing was conducted by Sanger ddNTP termination method using BrightDye reagent (Nimagen) and ABI 3130 Genetic Analyser (Applied Biosystems) at Far Eastern Federal University, Vladivostok. The sequences were assembled and edited using BioEdit (Hall, 1999). BioEdit was also used to extract the consensus sequences. The sequences used in this study, including those mined from GenBank, are listed in the Table 1.

**Table 1:** 16SrRNA nucleotide sequences used in this study.

Species	Location	Voucher #	GenBank #, 16S
*H. alexeyi*	Rudnaya Bay	*MIMB*–33243	KY464042
*H. djakonovi*	Rudnaya Bay	*MIMB*–33129	KY464038
*H. granulifera*	Vostok Bay	*MIMB*–33251	KY744471
*H. oculata*	Atlantic Ocean, UK	–	AY652500
*H. pachyderma*	Vostok Bay	*MIMB*–33252	KX610476
*H. sanguinolenta*	Norway	–	KT268115
*H. hayashii*	Rudnaya Bay	*MIMB*–33544	KY934074
*H. uluudax*	Starichkov Is.	*MIMB*–33543	KY934075
*H. uluudax*	Starichkov Is.	*MIMB*–33545	KY934076
*H. lineata*	Starichkov Is.	*MIMB*–33813	MF133322
*H. lineata*	Starichkov Is.	*MIMB*–33542/1	MF133323
*H. lineata*	Starichkov Is.	*MIMB*–33542/2	MF133324
*H. lineata*	Starichkov Is.	*MIMB*–33814	MF133325
*H. lineata*	Starichkov Is.	*MIMB*–33541	KY934077
*H. lineata*	Avacha Bay, Zavoiko Is.	*MIMB*–33812	MF133326
*H. lineata*	Mednyi Island, Bering Sea	*MIMB*–33115	KY934078
*H. olga* sp. n.	Rudnaya Bay	*MIMB*–33539	KY934079
*H. olga* sp. n.	Rudnaya Bay	*MIMB*–33540	KY934080
*H. olga* sp. n.	Rudnaya Bay	*MIMB*–33818/1	MF133327
*H. olga* sp. n.	Rudnaya Bay	*MIMB*–33818/2	MF133328
*H. olga* sp. n.	Rudnaya Bay	*MIMB*–33818/3	MF133329
*H. olga* sp. n.	Rudnaya Bay	*MIMB*–33818/4	MF133330

Two methods for species delimitation and identification were used: comparing tree topologies, and Automatic Barcode Gap Discovery (ABGD). The *p*-distances (i.e., the proportion of variable positions) and Neighbor-Joining (NJ) (Saitou & Nei, 1987) and Maximum Likelihood (ML) gene trees were calculated using MEGA 7 software (Kumar, Stecher & Tamura, 2016). Hasegawa-Kishino-Yano (HKY  + Γ + I) (Hasegawa, Kishino & Yano, 1985) evolutionary model was suggested by -lnL value found using Model Selection analysis implemented in MEGA. ABGD method (Puillandre et al., 2012) is based on pairwise distances, detecting the breaks in the distribution referred to as the “barcode gap” (Hebert et al., 2003) without any prior species hypothesis. The ABGD program is available at http://wwwabi.snv.jussieu.fr/public/abgd/abgdweb.html. I analyzed 16S alignment using either uncorrected *p*-distance or Kimura-2-Parameter (K2P) (Kimura, 1980) and Jukes-Cantor (JC) (Jukes & Cantor, 1969) distances. *X* (relative gap width) was set to 1.4, the other settings remained as default. Single pure (*SPu*) character attributes, i.e., species-specific barcoding positions (Sarkar et al., 2002; Sarkar, Planet & DeSalle, 2008; Bergmann et al., 2009), were detected manually because of low number of variable sites in *H*. cf. *spiculifera* group alignment.

The electronic version of this article in Portable Document Format (PDF) will represent a published work according to the International Commission on Zoological Nomenclature (ICZN), and hence the new names contained in the electronic version are effectively published under that Code from the electronic edition alone. This published work and the nomenclatural acts it contains have been registered in ZooBank, the online registration system for the ICZN. The ZooBank LSIDs (Life Science Identifiers) can be resolved and the associated information viewed through any standard web browser by appending the LSID to the prefix http://zoobank.org/. The LSID for this publication is: urn:lsid:zoobank.org:pub:398553F6-F96E-4B82-A6C5-366D7200AF0D. The online version of this work is archived and available from the following digital repositories: PeerJ, PubMed Central and CLOCKSS.

## Results

### Molecular analysis

Partial 16S rRNA nucleotide sequences obtained in this study and mined from GenBank were 597–609 b.p. long producing a 614 b.p. alignment, including gaps. Whole analyzed dataset included 101 variable, 510 conserved, and 60 parsimony-informative positions. The sequences of *H*. cf. *spiculifera* (i.e., *H. lineata, H. uluudax,* and *H. olga* sp. n.) included 10 variable/parsimony-informative, and 510 conserved sites. There is one (site #202: G for *H. lineata,* T for *H. uluudax,* and A for *H. olga* sp. n.) species-specific *SPu* character attribute for each of these three species, which allows to distinguish these species. *H. lineata* possesses two *SPu* (sites #424–C, #453–A), which barcodes this species against the other analyzed *Henricia* species, while *H. uluudax* possesses one such *SPu* (site #450–A). *H. olga* sp. n. does not possess any *SPu* character attribute, which may barcode it against the other analyzed species.

**Figure 2:**
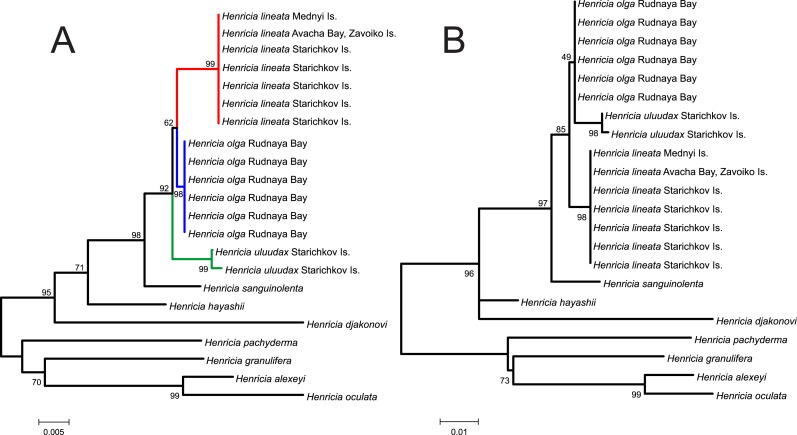
Species delimitation and phylogenetic reconstruction using the 16S rRNA marker. (A) Unrooted 16S NJ tree based on *p*-distances; (B) 16S ML tree of the subgenus *Setihenricia* rooted with four species of non-*Setihenricia* clades. Bootstrap indices at the internodes (1,000 replicates).

NJ/*p*-distance tree (Fig. 2A) shows well-supported delimited clusters for all studied species including the newly described species. Uncorrected interspecific distances ranged from 0.8–1.0% (*H. lineata–H. olga* sp. n., and *H. uluudax–H. olga* sp. n.) to 9.0% (*H. djakonovi–H. oculata*); the *p*-distance between *H. uluudax* and *H. lineata* was 1.5–1.6%. ABGD delimitation test using JC and K2P distances, revealed ten groups corresponding to all presumed species (prior maximal distance *P* = 0.001), although using the *p*-distances at *P* = 0.0129 failed to distinguish *H. sanguinolenta, H. lineata, H. uluudax,* and *H. olga* sp. n.; undoubtedly distinct species of the subgenus *Henricia, H. oculata* and *H. alexeyi,* also formed a single group.

Both NJ/*p*-distance and ML trees (Figs. 2A and 2B) demonstrate high bootstrap support (98% and 97%, respectively) for the relationship of *H. uluudax*, *H. lineata*, and *H. olga* sp. n. clade to *H. sanguinolenta*. Studied species with *H. sanguinolenta, H. hayashii,* and *H. djakonovi* form well-supported (96% ML and 95% NJ) subgenus *Setihenricia* clade. Relatively low bootstrap support of several other clades may be explained by short length of studied 16S fragment, which lacks phylogenetic signal hence these clades are not discussed here.

### Standardized descriptions of *Henricia* species

In this paper, I am using modified morphological description of *Henricia uluudax* by Clark & Jewett (2010) to elaborate a standard template for taxonomic descriptions of species the genus *Henricia*. For centuries, various authors have used different sets of characters and different terminology. In some cases, different terms were used for the same structure within one publication: e.g., Clark & Jewett (2010) used the terms ‘spine’, ‘spinelet’, ‘thorn’, and ‘spinule’ for a structure, which I call a ‘spine’ below, while a ‘thorn’ is a minor denticle on a spine. Such practice often obstructs side-by-side comparison of described entities belonging to this very diverse genus. To avoid this problem, I am proposing a standardized uniform description scheme and terminology based on a trade-off of the recent valuable publication by Clark & Jewett (2010) and keystone works by earlier authors. The main principle of this scheme is using strict consequence of described characters, verbatim overlaps in the descriptions of homologous characters, and using identical terms throughout all the descriptions in order to assist a reader to locate and compare the traits of interest. This template may and should be improved in the future by incorporating additional characters and terms.

## Systematics

**Table:** 

Order Spinulosida Perrier, 1884
Family Echinasteridae Verrill, 1870
Genus *Henricia* Gray, 1840
Subgenus *Setihenricia* Chichvarkhin & Chichvarkhina, 2017a
Type species: *Henricia hayashii* Djakonov, 1961

### *Henricia lineata* Clark & Jewett, 2010

*Henricia lineata* Clark & Jewett, 2010: 10–11, figs. 14–19; Jewett et al., 2015: 72–73.

*Henricia leviuscula spiculifera*—Verrill, 1914: 232 (part.), non *Cribrella spiculifera* Clark, 1901.

*? Henricia spiculifera*—Djakonov, 1950: 87 (part.), non *Cribrella spiculifera* Clark, 1901.

*Henricia* sp. A—R.N. Clark. www.jaxshells.org/henricia2.htm (accessed May, 2017)

*Henricia densispina*—Yavnov, 2010: 111–112, non *Cribrella densispina* Sladen, 1878.

Examined material: 1 spm, *MIMB*–33541, Starichkov Is, Kamchatka, 28 Jul 2015, leg. A Chichvarkhin; 5 spms, *MIMB*–33542, *MIMB*-33813-4, Starichkov Is, Kamchatka, 11 Aug 2016, leg. A Chichvarkhin; 1 spm, *MIMB*-33812, Avacha Bay, Zavoiko Is., Kamchatka, Aug 2015, leg. A Chichvarkhin; 2 spms, *MIMB*–33115, Mednyi Island, Bering Sea, Aug 2014, leg. N Sanamyan; 1 spm, Matua Is., Aug 2016, leg. N Sanamyan (examined by photo, preserved in ZIN).

**Figure 3:**
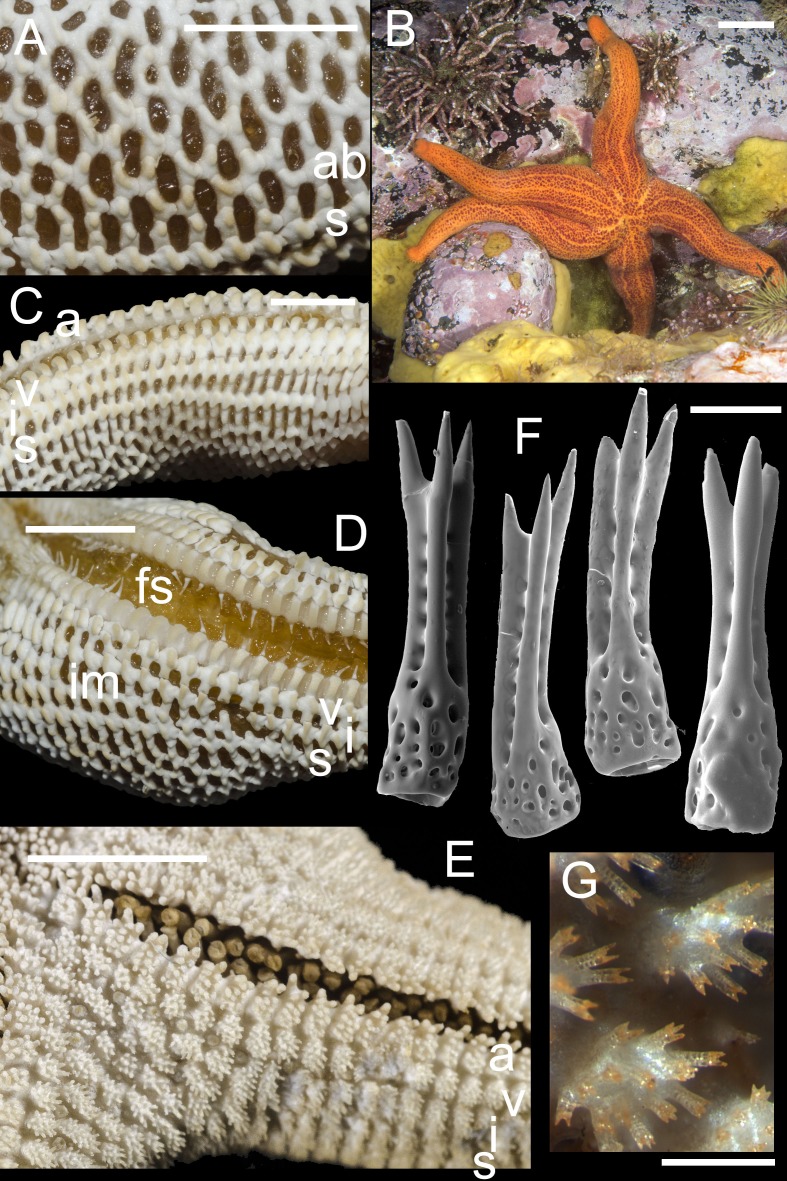
Henricia lineata. (A) abactinal plates; (B) living specimen, Starichkov Island, Avacha Bay; (C) marginal plates; (D) denuded actinal side; (E) actinal side of preserved specimen; (F) abactinal spines; (G) abactinal pseudopaxillae. a, adambulacrals; v, ventrolaterals; i, inferomarginals; im, intermarginals; s, superomarginals; ab, abactinal plates; fs, furrow spines. Scale bars: A–F-10 mm, F-50 µm, G-1 mm.

Average in size, R (long radius: mouth to ray tip) to 6.9, r (short radius: mouth to interradius) to 1.4 cm, *R*:*r* 5.1–6; disc small, rays moderately long, slender, subcylindrical, tapering to blunt tips. Abactinal surface (Figs. 3A and 3D) thick, semi-rigid; abactinal plates with high tubercles relatively small, forming regular mesh with no additional plates, forming tight reticulation; some plates very close set or fused into linear series, lacking papular areas between, forming three distinct lines on rays, similar linear series of plates at ray arcs, forming internal septa; plates crowned with 7–25 spines (7–15 on rays and 10–25 on disk) each tipped with three very sharp thorns (Figs. 3F and 3G); papular areas rather small, with 1–3 papulae; madreporite small, circular, spinose, located about half way between anus and edge of disc, at the end of the ray arc linear plate series. Superomarginals (Figs. 3A, 3C and 3D) slightly larger than abactinal plates bear 15–20 spines, inferomarginals about twice high as long; twice as large as superomarginal plates and bearing 25–30 thorn-tipped spines; a single series of intermarginals extends about 1/3 to 1/2 of R, each plate bearing 6–10 spines; ventrolateral series extending about 90% of R, a second series extending about 1/4 to 1/3 of R. Adambulacrals (Figs. 3D and 3E) each with a single deep furrow spine and 11–13 actinal spines, 3–4 longer and somewhat flattened spine(s) at furrow edge, and 9–10 smaller distally grading spines behind.

Oral plates with five marginal and 4–6 suboral spines; in addition, there are 2–4 thick, blunt, triangular teeth deep in the furrow near the distal edge of plate. Color in life (Fig. 3B), deep orange abactinally, with pale red to yellow-orange radial lines; orally yellow-orange. Preserved specimens retaining the lined pattern.

**Distribution**. Present at Commander Islands, Russia and along the Kamchatka and Kurile Islands. Also found throughout the Aleutians from Fox Islands Avatanak Island to Near Islands, Attu Island, Chichagof Harbor (type locality) at depths of 6–25 m.

### *Henricia uluudax* Clark & Jewett, 2010

*Henricia uluudax* Clark & Jewett, 2010: 11–14, figs. 21–26; Jewett et al., 2015: 84–85.

*Henricia spiculifera* (Clark) Verrill, 1914: 232 (part.), non *Cribrella spiculifera* Clark, 1901.

*Henricia leviuscula spiculifera*—Hayashi, 1940: 130–132, pl. 9, figs. 3–4 (part.?).

*Henricia leviuscula multispina* Fisher, 1911: 286 (part.), *non Henricia leviuscula multispina* Fisher, 1910: 571.

*Henricia spiculifera*—Djakonov, 1949: 29 (part.); Djakonov, 1950: 95 (part.); Djakonov, 1961: 15–16 (part).

Examined material: 2 spms, *MIMB*–33543, *MIMB*–33545, Starichkov Is, Kamchatka, Aug 2016, leg. A Chichvarkhin; 1 spm, Avacha Bay, Kamchatka, Aug 2016, leg. A Chichvarkhin; 1 spm, *ZIN*–33/15186, Bering Island, 1883, leg. Grebnitsky; 1 spm, *ZIN*–34/15210, 1 spm, *ZIN*–33/15186 Bering Island, 1883, leg. Grebnitsky; 1 spm, *ZIN*–31/15154, GEVO station 8, Sea of Okhotsk, 36 ftms, 13 Aug 1918, leg. Meder; 1 spm, Hokkaido University, labeled as *H. leviuscula spiculifera* f. *multispina*, collection data is unavailable.

**Figure 4:**
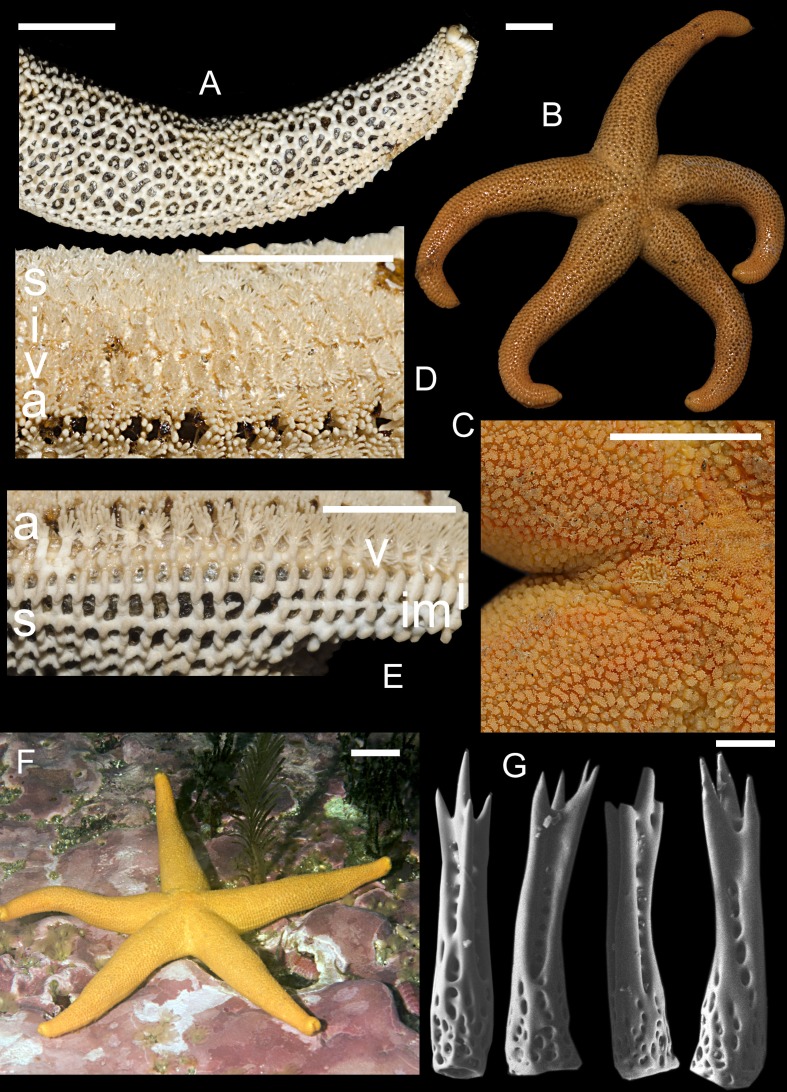
Henricia uluudax. (A) abactinal plates; (B) life coloration, Avacha Bay; (C) abactinal side of disk, live specimen; (D) actinal side of preserved specimen; (E) denuded actinal side; (F) living specimen, Avacha Bay; (G) abactinal spines. a, adambulacrals; v, ventrolaterals; i, inferomarginals; im, intermarginals; s, superomarginals; fs, furrow spines. Scale bars: A–F-10 mm, G-50 µm.

Average in size, *R* to 6 cm, *r* to 1.2 cm, *R*:*r* 4.7–5.3; disc small, rays moderately long, slender, tapering to fairly blunt tips (Fig. 4B). Abactinal plates small, with well-developed tubercles, numerous additional plates connected or not connected with other plates (Figs. 4A and 4B); pseudopaxillae form a tight reticulation, some apical plates often very close-set, nearly coalescing, forming three very fine lines at apex of ray; plates crowned with fine, divergent, thorn-tipped spines (Fig. 4G), 7–45 on rays and 25–50 spines on disk, about 0.25–0.30 mm in length; usually 3–4 slender thorns per spine, often, one thorn larger than others; papular areas rather small, with 2–3 papulae; madreporite small, circular, irregularly radially spinose, located about 1/3 of the distance between the anus and the edge of the disc. Superomarginals 1.5–2 times as large as abactinal plates, bearing 15–20 spines; inferomarginals about 2-fold larger than superomarginals, and bearing 30–35 thorny spines; first intermarginal series with 10–15 spines in pseudopaxillae about 1/2 as large as inferomarginals at base of rays, grading smaller aborally, extending about 40–50% of R, second series ending just pass the base of the rays; ventrolateral series bearing 25–35 spines on plate extending 75–80% of R. Adambulacrals (4) and (Figs. 4D and 4E) with a single deep furrow spine, and 13–16 actinal spines, four or five large, thick, blunt spines at edge of furrow, followed by a group of 10–13 smaller spines behind (usually arranged in 3 rows). Oral plates with 3–4 thick, blunt marginal spines, and 5–7 similar, suboral spines; in addition, there are 2–4 thick, blunt, triangular teeth deep in the furrow near the distal edge of the plate. Color in life (Figs. 4B and 4F) uniformly orange, red in Aleutian Islands; the lines of coalescing plates slightly darker than background. Preserved specimens may retain the lined pattern of coalescing plates, not in color.

**Distribution**. Found on the eastern coast of Kamchatka Peninsula, also in middle Kurile Islands (Matua Is.). A specimen from Hokkaido University (presumably obtained from Kurile Islands) also belongs to this species. Also, reported from Fox Islands, Avatanak Island to Andreanof Islands, Atka Island, Crescent Bay, point at W end at depths of 0–12 m.

**Remarks**. The sea stars obtained in this study are very similar to original description by Clark & Jewett (2010), although they described higher maximal number of spines in abactinal and superomarginal pseudopaxillae. Also, all sea stars examined and encountered by me in the wild possess bright to pale orange coloration, whereas the stars from the Aleutians were described as bright red. Two lateral lines of coalescing plates on the rays are not discernible in Aleutian specimens.

### *Henricia olga* sp. n.

urn:lsid:zoobank.org:act:1F56B99D-9998-4257-8F4C-6A04146C6359

*Henricia spiculifera*—Djakonov, 1958: 303, fig. 13 (part.); Djakonov, 1961: 15–16 (part.).

*Henricia leviuscula spiculifera*—Xiao, Liao & Liu, 2011: 12, figs. 8A–8F, 12C (part.?).

*Henricia* sp.—Chichvarkhin & Chichvarkhina, 2017a: fig. 4C; Chichvarkhin & Chichvarkhina, 2017b: fig. 1A.

Type locality: Rudnaya Bay, Sea of Japan, Russia.

Type material: Holotype (*R* = 35.5 mm, *r* = 6.3 mm) *MIMB*–33539, Rudnaya Bay, Sea of Japan, Russia, 5 Jun 2016; paratype (*R* = 16 mm, *r* = 3.0 mm) *MIMB*–33540, Rudnaya Bay, Sea of Japan, Russia, 5 Jun 2016.

Examined material: 1 spm, Rudnaya Bay, Sea of Japan, Russia, 5 Oct 2015; *MIMB*–33610, 4 spms, Rudnaya Bay, Sea of Japan, Russia, 1 May 2017; 3 spms, *ZIN*–4/14688, Toporok station 101, trawling SE from Iturup Is., 414 m, 14 Sep 1949; 1 spm, *ZIN*–30/15059, Toporok station 79, S from Shikotan Is., 50 m, 10 Sep 1949; 2 spms, *ZIN*–29/15058, Toporok station 2, Musasi Bank, Sea of Japan, 93–96 m, 16 Aug 1948; 1 spm, *ZIN*–59/15389, Toporok station 31, SW Sakhalin near Kaiwato, 84 m, 14 Aug 1949; 1 spm, *ZIN*–60/15570, Vityaz station 20, Sea of Okhotsk, 228–550 m, 1949. Also, at least five individuals were recorded by the author during 2013–2015 at Senkina Shpaka pinnacle (Rudnaya Bay) but were not preserved.

**Diagnosis**. Relatively small, fairly rigid, *R* to 4.8 cm, *r* to 1.0 cm; disc small, rays long, slim. Abactinal plates elongated, wide-set; some plates forming three well-discernible medial lines or ridge on the rays; abactinal spines slender with three long thorns. Superomarginal plates similar to abactinals, superomarginal row of pseudopaxillae poorly discernible. Adambulacral plates with 13–16 spines divided into two distinct groups: 5 stout near-furrow spines and 9–11 nearly three-fold shorter slender spines.

**Figure 5:**
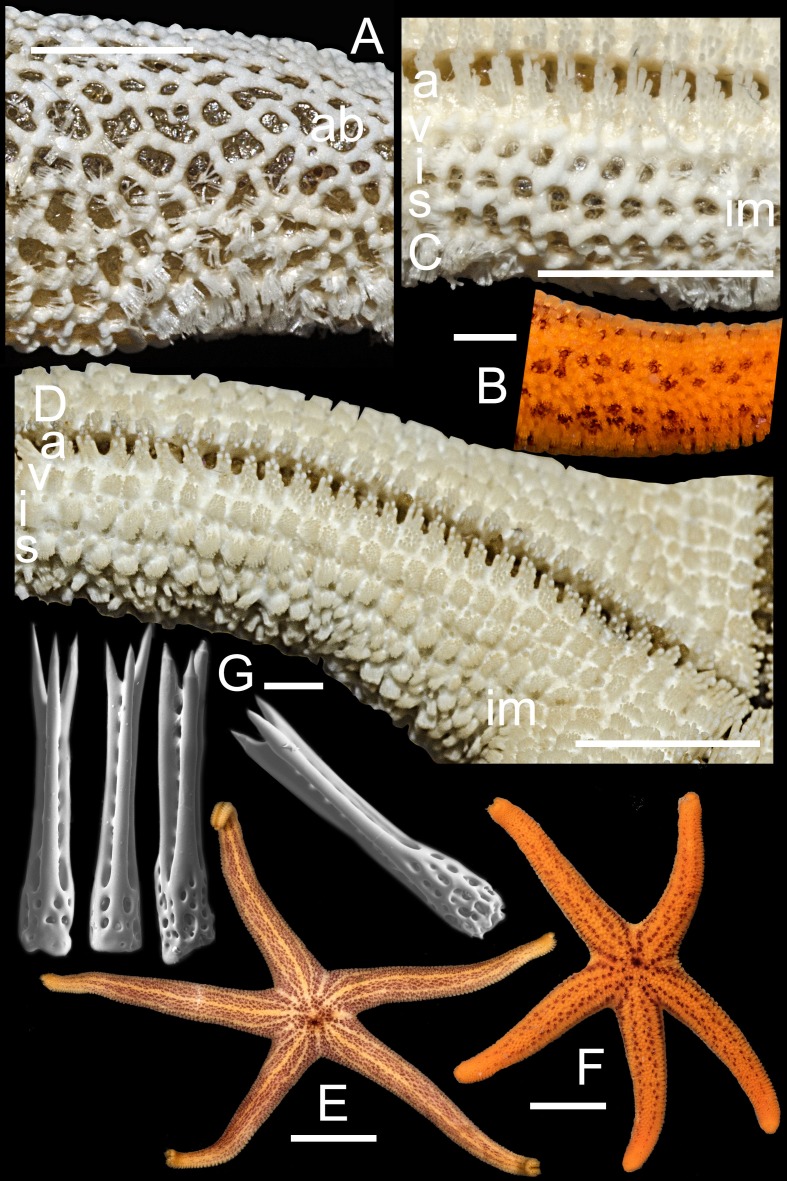
*Henricia olga.* sp. n. Holotype: (A) denuded abactinal plates, holotype; (B) abactinal side, living specimen, paratype; (C) denuded marginal plates, holotype; (D) actinal side of preserved specimen, holotype; (E) life coloration, holotype; (F) life coloration, paratype; (G) abactinal spines (scale bar 50 mkm). a, adambulacrals; v, ventrolaterals; i, inferomarginals; im, intermarginals; s, superomarginals; ab, abactinal plates. Scale bars: A, C, D, F-5 mm, B-1 mm, E-10 mm, G-50 µm.

**Description**. Relatively small, *R* to 4.8 cm, *r* to 1.0 cm, *R*:*r* 5.3–5.6; disc small, rays long, slender, tapering to blunt tips (Figs. 5E and 5F). Abactinal plates elongated, wide-set (Figs. 5A and 5B), with low tubercles, rare additional plates present connected with other plates; pseudopaxillae form a tight reticulation, some apical plates close-set or fused into linear series, lacking papular areas between, forming three well discernible lines or ridges at apex of ray; plates crowned with slender, divergent, thorn-tipped spines (Fig. 5G), 5–15 on rays and 25–35 spines on disk, about 300 µm in length; three slender thorns per spine; papular areas small, with 2–3 papulae; madreporite small, circular, irregularly radially spinose, located about 1/3 of the distance between the anus and the edge of the disc. Superomarginals as large as abactinal plates, bearing 15–20 spines, superomarginal row poorly discernible in non-denuded state; inferomarginals about 1/3 third larger than superomarginals, and bearing 30–35 thorny spines; first intermarginal series with 10–15 spines in pseudopaxillae about 1/2 as large as inferomarginals at base of rays, grading smaller aborally, extending about 40–50% of R, second series ending just past the base of the rays; ventrolateral series bearing 25–30 spines on plate extending 95% of R. Adambulacrals (Fig. 5D) each with a single deep furrow spine, and 13–16 actinal spines, arranged in two clearly distinct groups: 4-5 large, thick, blunt spines at edge of furrow, followed by a group of 9–13 three-fold shorter spines behind, arranged in three rows. Oral plates (Fig. 5D) with 3–4 thick, blunt marginal spines, and 2–3 suboral spines; in addition, there are two thick, blunt, triangular, teeth deep in the furrow near the distal edge of the plate. Color in life (Figs. 5E and 5F) striped: orange lines over pale brown background; juvenile paratype looks negative with dominating orange color. Preserved specimens possess almost indistinguishable the lined pattern.

**Distribution**: Found on the western coast of the Sea of Japan in Rudnaya Bay. Occurrence in the Yellow Sea, southern Kurile Islands and near western Sakhalin Island must be confirmed.

**Ecology**. The types were found on rocks at the depth of 15 m. In Sakhalin and the southern Kuriles found at 50–414 m depths.

**Etymology**. Female name Olga, common in Russia and northern Europe, which follows me throughout my life being shared by many of my good friends, colleagues, also by my wife, grandmother, and two aunts.

**Table 2:** Diagnostic characters of studied *Henricia* species.

	Additional abactinal plates	Life coloration	Abactinal spines	Adambulacral spines	Number of thorns in abactinal spines	Rays shape	Superomarginal pseudopaxillae	Inferomarginal plates
*lineata*	No	Longitudinally striped orange/brown, vivid colors	7–25	3–4 slightly flattened +9–10	3	Slim, cylindrical, blunt tips	1.5-fold larger than abactinals, with 15–20 spines	2-fold larger than superomarginals, with 25–30 spines
*uluudax*	Yes	Red, orange	7–50	4–5 + 10–13	4	Slim, cylindrical, blunt tips	1.5-fold larger than abactinals, with 15–20 spines	2-fold larger than superomarginals, with 30–35 spines
*olga*	Very rare	Longitudinally striped orange/grey, pale colors	5–35, very slender	3–5 very long + 9–11	3	Very slim, cylindrical	As large as abactinals, with 15–20 spines	1/3 larger than superomarginals, with 30–35 spines

## Discussion

The three examined sea star species can be well delimited and identified using the 16S molecular marker. However, low number of variable sites in their alignment suggests a discovery of additional markers with more robust barcode. These species form a monophyletic clade with *Henricia sanguinolenta* within the subgenus *Setihenricia.* Morphologically, these species are rather similar but possess several robust diagnostic traits listed in Table 2. Life coloration is probably the best character for their identification: vivid and striped in *H. lineata*, uniformly orange or red in *H. uluudax*, and pale striped in *H. olga* sp. n. Although *H. lineata* and *H. olga* sp. n. are similarly colored, the latter species possesses more slender rays, poorly discernible superomarginals, and southern distribution; *H. lineata* lacks additional abactinal plates. The adambulacral spines can be used to distinguish preserved *H. olga* sp. n. specimens by two sets of adambulacral spines: 3–5 near-furrow spines are stouter and 2–3-fold longer than the other spines, whereas in two other species, the size of adambulacral spines decreases almost gradually without a conspicuous “step”. *H. uluudax* can be easily distinguished by more numerous spines in aboral pseudopaxillae and stout four-thorned abactinal spines. Similar spines are a character of *H. densispina* but this species does not possess the lines of nearly-coalescing aboral plates extending along the rays. A similar species *H. multispina* was reported from Aleutian Islands (Jewett et al., 2015): its life coloration is pale orange, pale lavender or pale pink, its adambulacrals bear 35–50 spines, many more than in the other sympatric *Henricia* species, and it also lacks the rows of coalescing abactinal plates. *H. sanguinolenta* (Müller, 1776) may bear rather discernible rows of almost fused plates but it can be distinguished by more than 4 thorns on stout abactinal spines and by its pink to violet life coloration.

The specimens from Sakhalin Island and the southern Kuriles (Fig. 1, yellow pentagons) preserved in ZIN and identified earlier (mainly by AM Djakonov) as *H. spiculifera* and *H. multispina,* also the individual imaged by Xiao, Liao & Liu (2011) as *H. leviuscula spiculifera* are similar to *H. olga* sp. n. Although, the sea stars from Sakhalin and Kuriles possess stouter thick rays and discernible superomarginal series of the plates that are similar in size to the abactinals. Their life coloration is unknown. All of them share a near-furrow group of few very long spines and a group of 3-fold smaller ones as in *H. olga* sp. n. Therefore, a further study of these forms is necessary to reject or support a hypothesis about their belonging to *H. olga* sp. n.

##  Supplemental Information

10.7717/peerj.3489/supp-1 Supplemental Information 1 New nucleotide sequences, FASTA file, RAR archive

## Additional Information and Declarations

### DNA Deposition

The following information was supplied regarding the deposition of DNA sequences: The large subunit of mitochondrial rRNA partial sequences described here are accessible via GenBank accession numbers KY934074 –KY934080, KX610476, KY464042, KY464038, KY744471, MF133322 –MF133330.

### Data Availability

The following information was supplied regarding data availability: The raw data has been supplied as a Supplementary Dataset.

### New Species Registration

The following information was supplied regarding the registration of a newly described species: Publication LSID: urn:lsid:zoobank.org:pub:398553F6-F96E-4B82-A6C5-366D720 0AF0D. *Henricia olga*: urn:lsid:zoobank.org:act:1F56B99D-9998-4257-8F4C-6A04146C6359.

## References

[ref-1] Bergmann T, Hadrys H, Breves G, Schierwater B (2009). Character-based DNA barcoding: a superior tool for species classification. Berliner und Münchener Tierärztliche Wochenschrift.

[ref-2] Chichvarkhin A (2017). *Henricia djakonovi* sp. nov. (Echinodermata, Echinasteridae): a new sea star species from the Sea of Japan. PeerJ.

[ref-3] Chichvarkhin A, Chichvarkhina O, Dautova TN (2017a). A new sea star species of the genus *Henricia* Gray, 1840 (Echinodermata, Asteroidea) from the northwestern Sea of Japan and description of a new subgenus. Life-supporting Asia-Pacific marine ecosystems, biodiversity and their functioning.

[ref-4] Chichvarkhin A, Chichvarkhina O (2017b). Sea stars of the genus *Henricia* Gray, 1840 (Echinodermata, Asteroidea) from the northwestern Sea of Japan. Scientific and technological developments of research and monitoring of marine biological resources, May 22–24.

[ref-5] Chichvarkhin A, Chichvarkhina O, Wakita D (2017). First record of sea star *Henricia pachyderma* Hayashi, 1940 (Echinodermata, Spinulosida, Echinasteridae) in Russian fauna. Investigations of Aquatic Biological Resources of Kamchatka and the Northwestern Pacific.

[ref-6] Clark HL (1901). Echinoderms from Puget Sound, observations made on the echinoderms collected by the parties from Columbia University, in Puget Sound in 1896 and 1897. Proceedings of Boston Society of Natural History.

[ref-7] Clark RN, Jewett SC (2010). A new genus and thirteen new species of sea stars (Asteroidea: Echinasteridae) from the Aleutian Island Archipelago. Zootaxa.

[ref-8] Djakonov AM (1949). Key to the echinoderms of the Far Eastern Seas. Izvestiya TINRO.

[ref-9] Djakonov AM (1950). Sea stars of the seas of the USSR. Keys to the Fauna of the USSR.

[ref-10] Djakonov AM (1958). Echinoderms (Echinodermata), except holothurians, collected by Kuril-Sakhalin expedition in 1947–1949. Issledovaniya Delnevostochnykh Morei SSSR.

[ref-11] Djakonov AM (1961). Review of sea stars of the genus *Henricia* Gray from the northwestern parts of Pacific Ocean. Issledovaniya Delnevostochnykh Morei SSSR.

[ref-12] Fisher WK (1910). New starfishes from the North Pacific. II. Spinulosa. Zoologischer Anzeiger.

[ref-13] Fisher WK (1911). Asteroidea of the North Pacific and adjacent waters. Part 1. Phanerozonia and Spinulosa. Bulletin of the United States National Museum.

[ref-14] Fisher WK (1928). Asteroidea of the North Pacific and adjacent waters. Part 2. Forcipulata (Part). Bulletin of the United States National Museum.

[ref-15] Fisher WK (1930). Asteroidea of the North Pacific and adjacent waters. Part 3. Forcipulata (Concluded). Bulletin of the United States National Museum.

[ref-16] Gray JE (1840). A synopsis of the genera and species of the Class Hypostoma (Asterias Linn). Annual Magazine of Natural History, Series 1.

[ref-17] Hall TA (1999). BioEdit: a user-friendly biological sequence alignment editor and analysis program for Windows 95/98/NT. Nucleic Acids Symposium Series.

[ref-18] Hasegawa M, Kishino H, Yano T (1985). Dating of human-ape splitting by a molecular clock of mitochondrial DNA. Journal of Molecular Evolution.

[ref-19] Hayashi R (1940). Contributions to the classification of the sea-stars of Japan. I. Spinulosa. Journal of the Faculty of Science, Hokkaido Imperial University, Series VI: Zoology.

[ref-20] Hebert P, Cywinska A, Ball S, Waard J (2003). Biological identifications through DNA barcodes. Proceedings of the Royal Society B.

[ref-21] Jewett SC, Clark RN, Chenelot E, Harper S, Hoberg MK (2015). Field guide to sea stars of the Aleutian Islands.

[ref-22] Jukes TH, Cantor CR, Munro HN (1969). Evolution of protein molecules. Mammalian protein metabolism.

[ref-23] Kimura M (1980). A simple method for estimating evolutionary rate of base substitutions through comparative studies of nucleotide sequences. Journal of Molecular Evolution.

[ref-24] Kumar S, Stecher G, Tamura K (2016). MEGA7: molecular evolutionary genetics analysis version 7.0. Molecular Biology and Evolution.

[ref-25] Lambert P (2000). Sea stars of british columbia, southeast alaska and puget sound. Royal british columbia museum handbook.

[ref-26] Madsen FJ (1987). The *Henricia sanguinolenta* complex (Echinodermata, Asteroidea) of the Norwegian Sea and adjacent waters. A re-evaluation, with notes on related species. Steenstrupia.

[ref-27] Müller OF (1776). Zoologiae Danicae prodromus, seu Animalum Daniae et Norvegiae indigenarum characteres, nomina, et synonyma imprimis popularium.

[ref-28] Palumbi S, Hillis DM, Moritz C, Mable BK (1996). Nucleic acids II: the polymerase chain reaction. Molecular systematics.

[ref-29] Perrier E (1884). Mémoire sur les étoiles de mer recueilliés dans la mer des Antilles et le golfe du Mexique: durant les expéditions de dragace faites sous la direction de M. Alexandre Agassiz. Archives (Muséum National D’Histoire Naturelle (France)), 2.

[ref-30] Puillandre N, Lambert A, Brouillet S, Achaz G (2012). ABGD, Automatic Barcode Gap Discovery for primary species delimitation. Molecular Ecology.

[ref-31] Rasmunsen BN (1965). On taxonomy and biology of the north atlantic species of the asteroid genus *Henricia* gray. Meddelelser fra Danmarks Fiskeri-og Havundersøgelser. Ny Serie.

[ref-32] Saitou N, Nei M (1987). The neighbor-joining method: a new method for reconstructing phylogenetic trees. Molecular Biology and Evolution.

[ref-33] Sarkar IN, Planet PJ, DeSalle R (2008). CAOS software for use in character-based DNA barcoding. Molecular Ecology Resources.

[ref-34] Sarkar IN, Thornton JW, Planet PJ, Figurski DH, Schierwater B, DeSalle R (2002). An automated phylogenetic key for classifying homeoboxes. Molecular Phylogenetics and Evolution.

[ref-35] Shin S, Rho BJ (1996). Illustrated encyclopedia on fauna and flora of Korea.

[ref-36] Sladen WP (1878). On the asteroidea and echinoidea of the orean seas. Journal of the Linnean Society (Zoology).

[ref-37] Stimpson W (1857). On the crustacea and echinodermata of the Pacific shores of North America. Boston Journal of Natural History.

[ref-38] Verrill AE (1870). Notes on radiata in the museum of yale college. 1. Descriptions of new starfishes from New Zealand. Transactions of the Connecticut Academy of Arts and Sciences.

[ref-39] Verrill AE (1909). Remarkable development of starfishes on the northwest American Coast: hybridism; multiplicity of rays; teratology; problems in evolution; geographical distribution. American Naturalist.

[ref-40] Verrill AE (1914). Monograph of the shallow-water starfishes of the North Pacific coast from the Arctic Ocean to California. Harriman Alaska Series.

[ref-41] Xiao N, Liao Y, Liu R (2011). Records of the genus *Henricia* Gray, 1840 (Echinodermata: Asteroidea: Echinasteridae) from Chinese waters. Zootaxa.

[ref-42] Yavnov SV (2010). Atlas of the sea stars of the Far Eastern seas of Russia.

